# Psychosocial predictors of short-term glucose among people with diabetes: A narrative review

**DOI:** 10.1007/s10865-024-00536-9

**Published:** 2024-12-19

**Authors:** Fiona S. Horner, Vicki S. Helgeson

**Affiliations:** 1 Department of Psychology, Carnegie Mellon University, 5000 Forbes Avenue, Pittsburgh, PA 15213, USA

**Keywords:** Review, Glucose, Diabetes, Mood, Stress, Social interactions

## Abstract

Type 1 and type 2 diabetes are metabolic disorders that require one to manage one’s blood glucose levels on a daily basis through a series of behaviorally complex tasks. Research shows that psychosocial factors, including mood, stress, and social relationships, have a significant influence on one’s ability to maintain these disease management routines and achieve healthy blood glucose levels. However, researchers have typically approached these questions from a between-person perspective. Here, we argue for greater consideration of short-term, within-person links of psychosocial factors— including mood, stress, and social interactions—to glucose outcomes. Drawing from existing social and health psychology theories, we put forth an organizing theoretical framework describing how psychosocial experiences may operate on glucose outcomes over subsequent hours. We then review the small but burgeoning literature of intensive longitudinal studies that have examined the short-term effects of negative affect, positive affect, stress, and social interactions on glucose outcomes. Findings showed somewhat stronger links for negative affect and stress compared to positive affect and social interactions, but studies varied greatly in their methodologies, making direct comparisons challenging. A number of findings, particularly in the social interaction literature, depended on dispositional or contextual factors, further complicating interpretation. There was little investigation of the mechanistic pathways that may connect psychosocial factors to glucose outcomes, and few studies conducted lagged analyses to probe the directionality of these links. We conclude by proposing best practices for future research that will address the key weaknesses in the extant literature.

Type 1 and type 2 diabetes are behaviorally complex diseases each requiring a host of disease management behaviors in order to manually regulate one’s glucose levels. Type 1 diabetes (T1D) is an autoimmune disease that leads to the destruction of insulin-producing pancreatic cells, necessitating exogenous insulin administration (DiMeglio et al., 2018). Thus, those with T1D must frequently check their glucose values, monitor carbohydrate intake and energy expenditure, and adjust insulin doses and administration throughout the day based on these fluctuating factors. By contrast, type 2 diabetes (T2D) is characterized by insulin resistance and defective insulin secretion by pancreatic beta cells. Those with T2D may or may not check glucose regularly; treatment instead focuses on both pharmacological and behavioral interventions aimed at reducing cardiorenal risk and achieving glycemic and weight management goals (American Diabetes Association Professional Practice Committee et al., 2023; Galicia-Garcia et al., 2020). As T2D progresses, intensive insulin therapy (similar to T1D) is often required. Importantly, the behavioral tasks associated with both types of diabetes constitute lifelong regimens that persons with diabetes (PWD) must continually integrate into daily life so as to avoid a number of life-threatening microvascular and macrovascular complications (DiMeglio et al., 2018; Galicia-Garcia et al., 2020). Diabetes is thus a highly stressful disease and requires significant adjustment of one’s daily life.

It has long been recognized that psychosocial factors like mood, stress, and social relationships bear great influence over diabetes outcomes. The strongest evidence for these associations comes from studies that assess links of psychosocial factors to glucose; these typically examine HbA1c, a measure of one’s mean blood glucose over the prior two or three months. A large body of work has identified reliable links between psychosocial factors and HbA1c. For example, negative affect, particularly depressed mood, is a robust predictor of high HbA1c (Pouwer et al., 2013). Similarly, stress, especially diabetes-specific stress, has shown to be highly detrimental for disease outcomes, in some cases even eclipsing depressed mood as a predictor of glucose (Aikens, 2012; Fisher et al., 2010). One’s emotional state can also facilitate improved outcomes. Positive affect and related constructs have been shown to predict lower HbA1c and better self-care behaviors (Horner et al., 2023; Robertson et al., 2012), although this literature is small in comparison to work on negative affect. Finally, a large body of research has investigated the role of social relationships in the context of diabetes. While negative or conflictual relationships are generally related to a higher HbA1c, social support shows more mixed links to diabetes outcomes (Van Vleet & Helgeson, 2020; Wiebe et al., 2016). Support receipt by close others may be perceived as helpful but can also be perceived by PWD as intrusive or controlling (Kelly & Berg, 2021; Wiebe et al., 2016).

It is thus clear that social and emotional factors have significant implications for diabetes outcomes. However, the vast majority of this work has relied on between-person, often cross-sectional methodologies which compare predictors and outcomes across persons (e.g., people with depression have higher glucose than people without depression). From an intervention perspective, this is problematic because diabetes management does not occur at the between-person level. Rather, one’s diabetes self-management takes place on a daily, or even hourly basis. These behaviors must be continually integrated with and adapted to the ever-changing environment of daily life. Investigation of more granular, within-person links can help identify the real-time barriers and facilitators of diabetes self-care and could lead to more effective intervention strategies that better account for the daily nature of diabetes management.

The question of how psychosocial influences operate on diabetes outcomes within-person and in real time is not new. Several early studies manipulated stress in a lab setting and assessed subsequent changes in glucose levels. These studies found mixed results, with some showing stress increased glucose levels (Goetsch et al., 1990; Gonder-Frederick et al., 1990), while others did not (Gilbert et al., 1989; Naliboff et al., 1985). A limitation of experimental methods is that they lack the ecological validity necessary for determining the real-life significance of stressors in daily life for PWD. An alternative, more ecologically valid approach to this question is to use intensive longitudinal methods (e.g., daily diary or ecological momentary assessment [EMA]) to repeatedly sample both glucose and psychosocial events as PWD go about daily life. A collection of early studies largely focusing on stress used this approach, primarily among those with T1D (e.g., Aikens et al., 1994; Goetsch et al., 1994; Halford et al., 1990; Riazi et al., 2004), but used small samples and n-of-one analyses, rendering it difficult to draw clear conclusions from their findings.

In the decades since this early work, a number of methodological and technological advancements have supported greater investigation in this area. First, the technology for measuring and collecting glucose data has undergone immense development. Continuous glucose monitors (CGMs)—devices that insert into the outer layer of one’s skin and provide interstitial glucose readings every few minutes—now offer dense data on one’s real-time glucose levels. Although CGMs have been available for years, recent improvements in size, ease of use, and insurance coverage have led to increased usage, particularly among people with T1D (Karakus et al., 2023). The near-continuous data collected by CGMs offer considerable advantages over HbA1c (which assesses glucose over several months) or glucometers (“finger sticks,” or self-monitoring of blood glucose [SMBG], which capture isolated moments in time and may give biased data due to when measures are taken). CGMs offer novel opportunities to assess a wider range of glucose metrics at more granular levels than was previously possible. While CGMs can be burdensome (Tanenbaum & Commissariat, 2023), they have thus revolutionized the way in which researchers can collect glucose data.

Second, the feasibility of collecting intensive longitudinal psychosocial data has greatly increased given developments in personal technology (e.g., smartphones). Whereas participants once had to complete paper-and-pencil diaries or carry personal study devices on which to complete EMAs, smartphones now provide numerous digital tools for data collection and allow for better control over assessment timing and compliance monitoring. Thus, researchers are now able to efficiently collect data from far larger samples than were previously possible.

Finally, statistical advancements have facilitated appropriate analysis of these complex data. Whereas early studies analyzed each participant individually before aggregating results, researchers now have a host of multilevel modeling approaches that can examine within-person effects while accounting for nested data, autocorrelation of repeated measures, and randomly timed survey administration.

Collectively, these technological and methodological advancements have led to the development of a small but growing body of literature that has used intensive longitudinal methodologies to examine the short-term, within-person implications of psychosocial factors—beyond just stress—for glucose among PWD. However, this literature is small, with studies varying greatly based on timescale, glucose metric, psychosocial factor of interest, population, analysis plan, and theoretical background (or lack thereof). There is thus a need for a comprehensive survey of the literature to characterize our current understanding of these processes. The present review addresses this need. First, drawing from existing theories in social and health psychology, we discuss a theoretical model summarizing the paths through which psychosocial experiences can impact glycemia in real time. We then review empirical articles that have used intensive longitudinal methods (i.e., daily diary and EMA) to examine within-person links of mood, stress, and social interactions to glucose outcomes. We focus on these three categories of psychosocial predictors as they have received the most attention in the literature on psychosocial factors affecting diabetes health. In determining how these factors impact glucose outcomes across short timescales, we hope to better understand the direct impact of daily fluctuations in one’s social and emotional environment on one’s disease state. We then integrate these findings, highlighting key takeaways, identifying common weaknesses, and proposing best practices for this nascent literature.

## Theoretical framework

How exactly could mood, stress, or social interactions get under the skin to influence glucose? Two general paths—a physiological and a behavioral path —have long been recognized to account for this link (Hackett & Steptoe, 2017). However, theory in this area is often underspecified and fails to identify both the timescale at which such links occur as well as how these paths operate across distinct psychosocial phenomena. Here, we summarize the physiological processes that may underlie these links. Then, drawing from Self-Determination Theory (Deci & Ryan, 2011), we outline likely mechanisms underlying the behavioral pathway. Finally, we synthesize these processes into an organizing theoretical framework that, based on the stress buffering hypotheses of social support (Cohen & Wills, 1985) and positive affect (Pressman & Cohen, 2005), clarify the distinct processes that may differentially link varying psychosocial phenomenon to glucose outcomes. This framework will be useful both in the interpretation of the extant literature as well as in guiding future lines of research.

The first primary pathway linking stress to glucose is physiological. Epinephrine, released from the adrenal glands as part of the sympathetic nervous system’s immediate response to stress, elevates glucose levels by both increasing glucose production and inhibiting endogenous insulin secretion (Hackett & Steptoe, 2017; Surwit et al., 1992). Soon after this SNS response, the HPA axis activates, triggering a cascade of hormonal changes that ultimately results in the release of cortisol and other glucocorticoids. These hormones are central in maintaining glucose homeostasis and act by mobilizing energy stores while suppressing insulin secretion, resulting in raised glucose (Hackett & Steptoe, 2017; Surwit et al., 1992). Both of these processes onset soon after an acute stressor and can last several hours (Hannibal & Bishop, 2014). While these neuroendocrine responses are typically adaptive, they can be problematic in the context of diabetes where insulin deficiency and insulin resistance disrupt the return to glucose homeostasis. Long-term activation of autonomic and inflammatory processes has further implications for hyperglycemia, insulin resistance, and the development of T2D (Hackett & Steptoe, 2017). Given the current focus on short-term links we do not focus on these processes here, we but note that this may indicate an interaction between chronic and acute stress in predicting momentary glucose.

The second primary pathway is through the disruption or facilitation of diabetes management behaviors. Self-determination theory (SDT) is a cohesive theory of human motivation that identifies three basic psychological needs—autonomy, competence, and relatedness—as necessary for optimal functioning and intrinsic motivation (Deci & Ryan, 2011). Autonomy refers to feeling like one has control over one’s own choices and behaviors; competence refers to feeling that one is capable of the tasks at hand, and relatedness refers to having close social ties and feelings of social belonging. When these three needs are thwarted in some way—when a self-care failure challenges competence, a conflict with a spouse threatens relatedness, or a controlling parent undermines autonomy—one’s motivation is negatively impacted, which, in the context of diabetes, can impede one’s ability to maintain the numerous and continuous self-care behaviors demanded by the disease. Conversely, fostering these needs through supportive or positive experiences can support motivation surrounding one’s self-care. SDT has garnered empirical support across numerous domains, including health behaviors (Deci & Ryan, 2011).

**Fig. 1:**
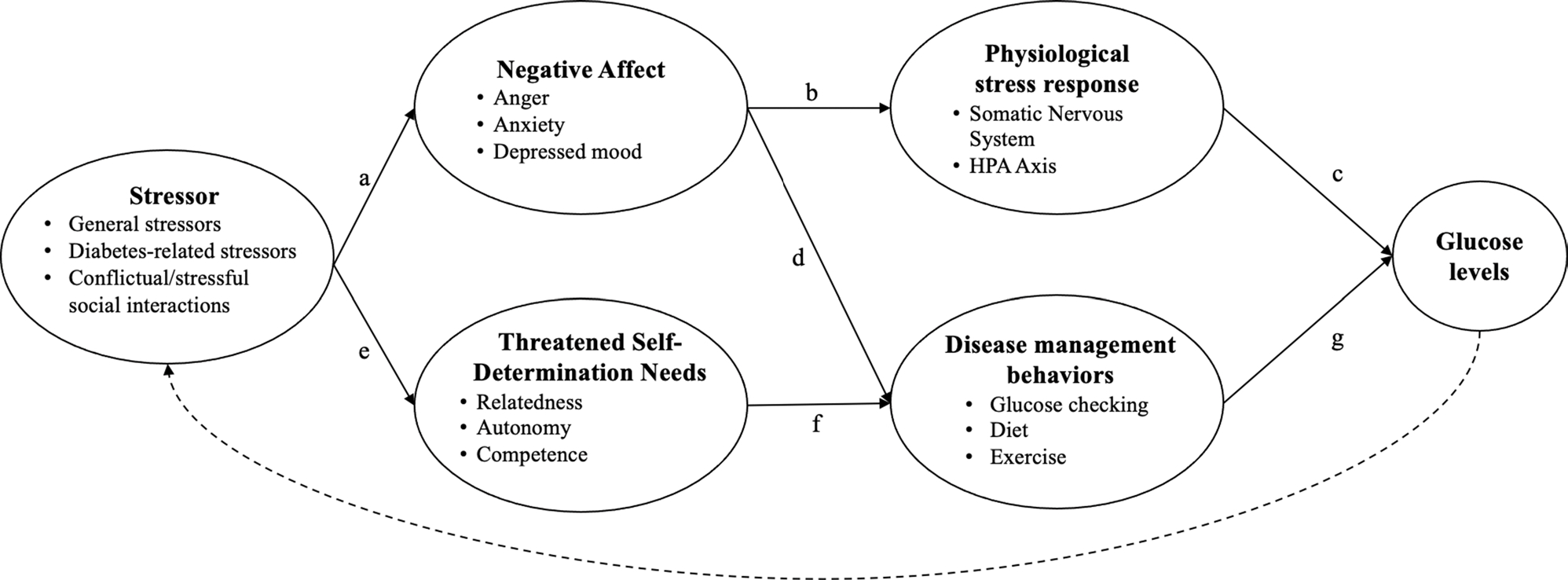
Pathways linking stressful or negative experiences to glucose

While these behavioral and physiological pathways are thus likely links between psychosocial factors and glucose, there has been little consideration of how these processes play out across distinct psychosocial experiences. In particular, the effect of stressful or conflictual experiences is likely distinct from that of positive or supportive experiences. As discussed above and depicted in Fig. 1, a stressor triggers negative affect (path a), which activates neuroendocrine responses that release epinephrine and cortisol (path b), ultimately elevating glucose levels (path c). Negative affect may additionally distract from one’s disease management regimen, thus disrupting glucose through the behavioral pathway (path d). At the same time, to the extent that a stressor challenges one’s needs of competency, autonomy, and relatedness (path e), it can interrupt one’s ability to maintain one’s diabetes self-care regimen (path f), again leading to glucose dysregulation (path g). Thus, a conflictual or stressful interaction has a direct influence on negative affect and self-determination needs and then activates physiological and behavioral mechanisms that ultimately influence glucose.

**Fig. 2:**
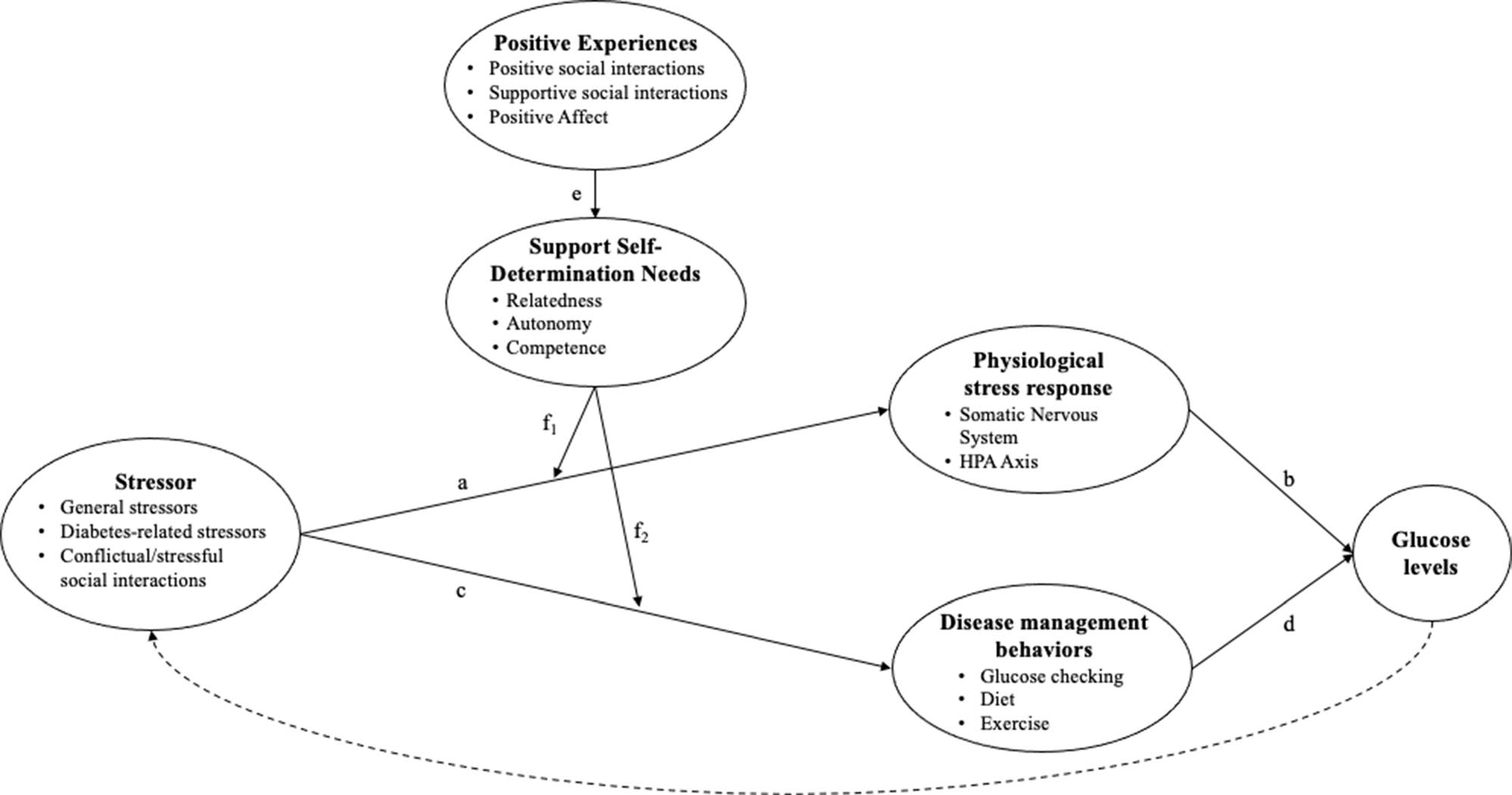
Pathways linking positive experiences to glucose

By contrast, the effect of positive experiences on glucose is likely less direct. While positive experiences span several distinct constructs, their hypothesized influences in the proposed model align; they are thus visualized together in Fig. 2. Here, we conceptualize positive experiences as the interrelated and co-occurring experiences of supportive social interactions (i.e., receiving help or support from close others), positive social interactions (e.g., having fun with others), and positive affect.

Considering first the physiological path outlined above, it is not clear that such positive experiences would simply wield the inverse effects to those outlined for negative experiences. Indeed, while some studies link positive affect to lower cortisol, epinephrine, and norepinephrine, other studies find the opposite link, or no link at all (Pressman & Cohen, 2005). The same has been found of social support. For example, a review of peer relationships among youth with diabetes found that friend support was typically unrelated to glucose levels, with some evidence that it was actually linked to higher glycemic outcomes (Van Vleet & Helgeson, 2020). Instead, drawing on the stress-buffering hypotheses of positive affect (Pressman & Cohen, 2005) and social support (Cohen & Wills, 1985), we propose that such positive experiences would primarily be beneficial in the context of stress (see Fig. 2). That is, if physiological stress processes are not already in action at a given moment (path a), a positive experience may be unrelated to subsequent short-term changes in glucose. However, once a stressor is perceived and these neuroendocrine processes are in motion, positive experiences, to the extent that they support the self-determination needs (path e), may help to attenuate the negative effects of stress (moderating path f_1_). For example, supportive social interactions increase feelings of relatedness, competence, and perceptions of one’s available social resources, thus helping the PWD reappraise the stressor as less threatening and reducing physiological stress responses. The positive emotions that accompany supportive and more generally positive social interactions may further act by reducing physiological stress reactivity or quickening recovery. Indeed, following a stressor, positive affect has been shown to facilitate faster recovery or smaller changes in physiological metrics related to stress, including heart rate variability, heart rate, and blood pressure (Pressman et al., 2018; Pressman & Cohen, 2005). Thus, in the context of an acute stressor, positive experiences may serve to dampen physiological stress responses (path f_1_) and subsequently reduce or avoid short-term rises in glucose levels (path b).

It should be noted that over long periods of time, both positive affect and positive social experiences exert main effects on many aspects of health (Cohen, 2004; Pressman et al., 2018); given our focus on immediate, within-person links, we do not discuss these processes here.

Concerning the behavioral pathway, positive experiences may facilitate self-care behaviors to the extent that they enhance motivation by increasing autonomy, competence, and relatedness. Indeed, research has linked positive affect and social support to health behaviors like physical activity, sleep, and medication adherence (Pressman et al., 2018). But at the same time, positive affect and positive experiences can interfere with daily self-care if they distract one from one’s disease, or if the desire to blend in and not draw attention to oneself in social situations leads one to consciously avoid self-care tasks (Mulvaney et al., 2019; Van Vleet & Helgeson, 2020). Again, approaching this from a stress-buffering perspective may help us better understand these conflicting paths. In the context of a stressor (Fig. 2, path c), the increased perceptions of relatedness, competence, or autonomy that stem from positive experiences (path e) may help the PWD cope with or reappraise the stressor at hand (moderating path f_2_). As a result, motivation regarding one’s diabetes care can be maintained, and the negative repercussions of the stressor on glucose through health behaviors can be attenuated or avoided (path d). Thus, the effect of positive experiences on glucose levels likely operates primarily through attenuating the stress-glucose link, rather than exerting direct influence on glycemia.

Of note, these processes operate in the context of one’s broader psychosocial environment, meaning that negative and positive experiences can co-occur. We present them separately so as to more clearly communicate our distinct hypotheses. Additionally, it should be noted that glucose levels can have further implications for one’s psychosocial experiences, as represented by the dashed lines in Figs. 1 and 2. For example, experiencing symptoms or seeing unexplained high glucose numbers can impact one’s mood and stress levels. This path is not our present focus, so we do not expand upon it here.

It should also be underscored that T1D and T2D are distinct diseases with different pathophysiologies; thus, these theorized links may play out differently across these two conditions. In particular, T1D—typically characterized by absolute insulin deficiency—results in greater glucose variability and higher risk for dangerous low glucose events. If no endogenous insulin is available when physiological stress elevates glucose values, then a single missed meal or missed insulin bolus will have significant and immediate effects on glucose. Thus, in T1D, psychosocial factors may impact glucose more quickly, regardless of the mechanistic pathway at play, and metrics of glucose variability and time below range are important outcomes to assess. By contrast, glucose levels in T2D are comparatively less variable, with a lower chance of having a severe low glucose event. In T2D, it is more likely that the body still produces some insulin which may attenuate the impact of physiological stress on glucose. Additionally, intensive insulin therapy is less common in T2D, and there is greater emphasis on fixed-dose oral medications. Thus, in T2D, psychosocial factors may operate on glucose over somewhat longer timescales and may have the largest effects on outcomes like glucose mean and time above range.

Given these theoretical links between varied psychosocial experiences and glucose outcomes, we now turn to the extant literature to characterize existing findings in this space.

## Literature search

Web of Science, PsychInfo, and Pubmed were searched from inception through January 2023. Articles were included in the initial search if the abstract included the term “diabetes” along with any one of a series of mood, relationship, or stress terms, any one of series of glucose terms, and any one of a series of terms indicating intensive longitudinal designs. Additional keyword and reference list searches were conducted in November 2023 and July 2024. See supplement for complete search terms.

The article screening process is summarized in Supplementary Fig. 1. The initial search resulted in 1655 articles; after removing duplicates, the total number was 986. Articles were included if they (a) were conducted among people with T1D or T2D, (b) employed an intensive longitudinal design in an ecologically valid context (e.g., daily diary, EMA, etc.), (c) measured glucose repeatedly within-person using either a CGM or SMBG (d) measured mood, stress, or an aspect of social interactions repeatedly within-persons, and (e) reported within-person relations of either mood, stress, or social interactions with glucose either cross-sectionally or in the direction of the psychosocial predictor predicting glucose. Articles were excluded if they were in a language other than English, if they used n-of-one analyses, or were conducted among non-human samples. Studies were not restricted based on the glucose summary metric used (e.g., glucose mean, standard deviation, etc.). The first author screened all articles. If an article’s eligibility was unclear, the second author was consulted. Studies were most commonly excluded because (1) they did not employ an intensive longitudinal methodology, or (2) they were not conducted among people with T1D or T2D. Fifteen studies met the inclusion/exclusion criteria from the database search. Additional keyword and reference list searches identified 13 more studies that met criteria. Thus, a total of 28 studies were included in the present review.

Because our theoretical perspective is concerned with the effect of psychosocial factors on glucose, we include only findings in which the psychosocial variable and glucose were assessed at the same time (i.e., contemporaneous findings) or in which glucose at one timepoint was regressed onto a psychosocial variable at a prior timepoint (either through statistical lagging or as the result of different measurement time frames). Findings from studies that examine the psychosocial variable at one timepoint regressed onto glucose at a prior timepoint are outside of the scope of this review.

We have organized the review first in terms of the three psychosocial predictor variables: mood/affect, stress, and social interactions. Because negative and positive affect represent distinct constructs (Diener & Emmons, 1984), we separately present findings for each. Within each predictor, we differentiate findings by timescale. Specifically, we divide findings into *daily* and *within-day* (e.g., hourly or momentary) categories. It should be noted that some studies assess multiple psychosocial predictors (e.g., positive and negative affect, or stress and social interactions) as well as daily and within-day links and are thus included in multiple sections. We differentiate studies among people with T1D versus T2D because each disease involves unique care regimens, stressors, and patterns of glucose fluctuation.

The identified articles used a wide range of glucose metrics. Most commonly, studies examined glucose mean, standard deviation (SD), coefficient of variation (CV), and times in ranges. Following national guidelines, we define time in range (TIR) as time spent with glucose levels between 70 and 180 mg/dL, time above range (TAR) as time with levels above 180 mg/dL, and time below range (TBR) as time with levels below 70 mg/dL (Danne et al., 2017). In some cases, the studies reported here diverged from these cutoffs, in which case we specify the glucose range evaluated.

## Literature review

### Negative affect

Studies on the association between negative affect and glucose are summarized in Table 1.

#### Daily

##### T1D.

Five studies have examined daily links of negative affect to glucose outcomes. Of these, three found support for the link, one found partial support, and the fourth did not support the link. One study found that, with a few exceptions, a series of negative mood items were each independently linked to lower TIR and higher TAR in an adult sample (Polonsky & Fortmann, 2020). Negative mood was not associated with CV or TBR, with the exception of “exhausted” being associated with greater CV. It should be noted in this study, however, that many of these items overlap with energy or fatigue, which are likely consequences—rather than predictors—of glucose excursions. Indeed, the authors approached these associations from the perspective of glucose predicting mood but evaluated this question in cross-sectional analyses which do not speak to the direction of these links.

Two studies conducted among adolescents align with these findings. The first found that on days when participants reported greater negative affect, they had higher glucose mean (Fortenberry et al., 2009). However, lagged analyses found no link for negative affect predicting next-day glucose or glucose predicting next-day negative affect. Thus, the directionality of these findings could not be determined. Another study found that on days in which adolescents experienced greater negative affect related to their diabetes they had higher glucose mean and SD (Lansing et al., 2017).

A small (*N* = 18) study among adults showed more mixed findings (de Wit et al., 2023). Overall, daily glucose CV was unrelated to mean or CV of participants’ negative mood. However, on days following a nocturnal hypoglycemic event, glucose CV was associated with higher mean levels of anxiety, depressed mood, and fatigue, and greater anger CV. Additionally, on days when glucose CV was greater than 36%, fatigue CV was higher. Again, the overlap of these mood measures with energy makes interpretation more difficult and is more in line with the reverse temporal direction. Finally, another study among adolescents found no link of daily negative affect to daily glucose CV (Shapira et al., 2021).

##### T2D.

Only one study has examined daily links of negative affect and glucose and did so using a lagged design. Results showed that negative affect on one day predicted increased fasting glucose the following morning (Skaff et al., 2009). This finding held even after a number of additional covariates, including depressive symptoms, were added to the model. There was no evidence for an effect in the opposite direction, i.e., glucose on one day did not predict the next day’s negative affect. These findings are particularly powerful given they are lagged. However, exploratory analyses showed that this finding was moderated by gender, such that negative affect predicted next-day glucose only among men.

##### Summary.

Collectively, these studies provide tentative support for a daily link of negative affect to glucose, although there is some evidence that negative affect is less impactful for glucose variability (e.g., CV) compared to mean glucose levels. The two studies that assessed lagged links showed mixed findings, with one finding negative affect predicted higher next-day glucose, while the other found no link. However, these studies differed in both participant age (adolescent/adult) and disease type (T1D/T2D), which may account for these differences. Finally, there is preliminary evidence that gender my influence the relations in question.

#### Within-day

##### T1D.

Four studies have examined within-day links of negative affect to glucose and provide somewhat weak evidence for this association. In a short (*M* = 48.8 h) EMA study, momentary tension, but not anger, was linked to current glucose values (Hermanns et al., 2007). A second study found that momentary ratings of anger—but not anxiety, depressed mood, or fatigue—were associated with above range glucose values (de Wit et al., 2023). Both of these studies approached these links from the perspective of glucose predicting mood but conducted cross-sectional analyses. Among teens, one study found that momentary negative affect was linked to very high glucose; this link was driven by those with low versus high HbA1c (Shapira et al., 2021). Negative affect was also marginally linked to *reduced* odds of very low glucose levels. Thus, this study found that negative affect is linked to higher glucose, which, while detrimental for long-term outcomes, may have the incidental benefit of reducing dangerously low glucose levels on a more immediate basis. Finally, a fourth study found no link between negative affect and glucose over the next two hours among an adolescent sample (Horner & Helgeson, 2024).

##### T2D.

Two studies have examined within-day links of negative affect to glucose. One study was concerned with whether glucose predicted mood and found that current glucose was not associated with current negative affect. However, a quadratic term for current glucose was associated with momentary negative affect such that both high and low glucose were linked to higher current negative affect (Williams et al., 2002). The second showed no relation between negative affect (measured morning and night) and glucose over the next 10 h (Wagner et al., 2017).

##### Summary.

There is thus weak evidence for within-day links of negative affect to glucose. There is some evidence that HbA1c may again impact these links. Findings also support investigation of quadratic links of glucose to negative affect; assuming a linear relation may obscure more complex underlying patterns. That is, negative affect may be linked to out-of-range glucose values broadly—including both high and low values—rather than simply higher glucose. Use of metrics like TIR, which captures both high and low excursions, may be more informative and interpretable in future research.

### Positive affect

Studies on the association between positive affect and glucose are summarized in Table 1.

#### Daily

##### T1D.

Four studies assessed daily links of positive affect to glucose. Two of these studies supported this link, while the remaining two offered more qualified findings. One study found that a series of distinct positive affect items were nearly all individually associated with greater TIR and less TAR in an adult sample (Polonsky & Fortmann, 2020). No links were found between the positive items and glucose CV or TBR. While the authors approached these relations from the direction of glucose predicting mood, these findings are contemporaneous. Similarly, another study found that positive affect was associated with lower same-day glucose among adolescents (Fortenberry et al., 2009). Mirroring the negative affect findings from this study, lagged analyses did not reveal the directionality of these links: positive affect did not predict next-day glucose, and glucose did not predict next-day positive affect.

A third study found no link of daily mood (mean or CV) to glucose CV (de Wit et al., 2023). However, on days following a nocturnal hypoglycemic event, glucose CV was associated with lower mean vigor and greater vigor CV. Additionally, on days in which glucose CV was greater than 36%, participants showed greater vigor CV. Noted that this study included a very small sample (*N* = 18).

Finally, a fourth study failed to find a link of positive affect to glucose CV among teens (Shapira et al., 2021). However, the authors noted that a marginal association emerged only among participants with HbA1c <8%. In this small subgroup (*N* = 9), days in which participants reported higher positive affect trended towards having lower daily glucose CV.

##### T2D.

Only one study has examined daily links of positive affect to glucose and did so using a lagged study design. This study found that, controlling for negative affect, within-person positive affect did not predict next-morning fasting glucose. Reverse-lagged analyses similarly failed to find a link of glucose on one day to positive affect the next day (Skaff et al., 2009).

##### Summary.

There is thus tenuous evidence at the daily level for a link between positive affect and healthier glucose outcomes among people with T1D, but the causal direction is unclear, and more work is needed. It may also be beneficial to examine the impact of other disease factors, like HbA1c or nocturnal hypoglycemia, on this link. By contrast, there is no evidence for this link among people with T2D, with only a single study examining this question among this population.

#### Within-day

##### T1D.

Four studies have assessed within-day links of positive affect to glucose. Two found evidence in support of this link, one showed that the link was complicated by HbA1c, and one found no evidence. The first examined “hedonic tone,” a combined measure of low sadness and high happiness, and “energetic arousal,” a measure of high-arousal positive mood, and found both measures were linked to lower momentary glucose values (Hermanns et al., 2007). Similarly, the second study found that above range momentary glucose values were associated with lower momentary vigor (de Wit et al., 2023). It should be noted that both of these studies used positive affect measures that overlap with energy level and may thus be a consequence rather than a predictor of glucose. Indeed, both studies approached these relations from the direction of glucose predicting mood but conducted contemporaneous analyses.

The third study provided weaker evidence, finding that positive affect was associated with more in-range glucose readings and fewer very low glucose readings only among adolescents with HbA1c < 8% (Shapira et al., 2021). Thus, in this sample positive affect was protective only for those with HbA1c close to the clinical target for this age group (≤7.5%; American Diabetes Association, 2021). A final study among adolescents showed no link of positive affect at one time point and glucose levels over the next two hours (Horner & Helgeson, 2024).

##### T2D.

Two studies have examined within-day links of positive affect to glucose; both failed to support this link. The first approached this question from the theoretical orientation of glucose predicting mood but did so using a cross-sectional methodology. Findings showed current glucose values as well as a quadratic glucose term were not related to current positive affect (Williams et al., 2002). The authors also investigated neuroticism as a moderator of these links; no evidence of moderation was found. The second study found no links between positive affect at one time point and glucose over the following 10 h (Wagner et al., 2017). Importantly, this study included positive affect, negative affect, and self-care behaviors as predictors in the same model. This null finding should thus be interpreted as positive affect having no additional effect on glucose beyond those of negative affect and self-care.

##### Summary.

Collectively, these studies point to tentative support for a within-day link of positive affect to glucose among people with T1D but not among people with T2D. Four of the five studies used cross-sectional, momentary measures of both mood and glucose which do not speak to the direction of these links. The fourth study used a lagged design and failed to find evidence for an effect of positive affect on subsequent glucose. Of note, several studies used measures of positive affect that are somewhat confounded by energy level (e.g., ‘vigor’, ‘energetic arousal’), complicating interpretation of these links.

### Stress

Here we review the literature linking stress to short-term glucose outcomes. We include studies that examined general stress (e.g., general perceived stress), diabetes-specific distress (i.e., stress related to the emotional, interpersonal, or regimen difficulties associated with diabetes), and diabetes problems (e.g., self-care failures, unexplained glucose values). Of note, our review only returned studies at the daily level among people with T1D; there were no within-day studies, and no studies among those with T2D. These studies are summarized in Table 2.

#### Daily

##### T1D.

Six studies have linked daily stress to daily glucose. These studies largely converge on the conclusion that daily diabetes-related stress is associated with worse glucose outcomes, but studies find less evidence for an effect of general stress.

One study showed that days with more diabetes stressors had higher daily glucose mean, but there was no link between the number of general daily stressors and glucose (Berg et al., 2020). ^1^ However, there was a significant cross-level interaction for general stressors, indicating that they were associated with higher glucose for those with greater comorbidities and lower glucose for those with fewer comorbidities, although neither slope was statistically significant. Age did not moderate the stressor-glucose link. Another study from the same lab group aligns with these results and found that daily diabetes stressors predicted higher same-day mean glucose, but daily general stressors did not (Berg et al., 2023).^2^ The authors also investigated whether general stress interacted with diabetes-specific stress to predict glucose; no evidence for moderation was found. Similarly, a recent dissertation found that days with more diabetes-specific stressors had higher daily mean glucose, but not daily glucose SD (Benjamin, 2021). While the author predicted stronger links for those higher in trait anxiety, no such interaction effect was found. Daily general stressors were unrelated to either glucose outcome.

In another study among adults recently hospitalized for diabetes complications, links of glucose to four different types of diabetes distress were examined. Results showed that on days when participants reported greater hypoglycemia distress, they had greater TBR, less TAR, and greater glucose CV. On days when participants reported greater hyperglycemia distress, they had less TBR, greater TAR and greater glucose CV, and on days when they reported greater glucose variability distress, they had greater TAR and greater glucose CV (Ehrmann et al., 2022). There were no links for general diabetes distress.

Two studies from the same small (*N* = 33) sample found more mixed evidence. The first assessed links between a single daily general stress item and a set of ten different daily glucose outcomes (Gonder-Frederick et al., 2016). Results showed that days in which PWD reported greater stress were marked by greater TBR and greater glucose variability as measured by glucose SD, daily risk range, and glucose rate of change standard deviations. However, there were no links for the other six outcome measures, which assessed both central tendency and variability of glucose. It should also be noted that these analyses relied on an ANCOVA and did not differentiate within- and between-person variances, meaning that some of these finding may be due to person-level differences rather than daily fluctuations. A later paper analyzed the same data using a multilevel framework and expanded on the prior work by testing interactions with BMI (Ozaslan et al., 2018). Results showed no link between daily stress and same-day glucose, regardless of one’s BMI. However, stress did predict a glucose effectiveness index; this finding was moderated by BMI such that stress had a smaller influence on the effectiveness index among those with a higher BMI.

##### Summary.

Collectively, there is evidence that diabetes distress and glucose are positively related on the daily level; there is less evidence for an effect of general stress. However, little work has attempted to tease apart the directionality of these findings, and the only study that examined lagged findings failed to find an association. Finally, it is a notable limitation that no studies examined within-day links of stress to glucose, and no studies examined links at any level among people with T2D.

### Social interactions

Given the many aspects of social interactions that could bring to bear on short-term glucose outcomes, this section is the most varied in terms of the independent variable. Our search identified studies that examined the affective quality of interactions, instances of direct involvement and tangible support received for one’s disease, and information shared during an interaction. Studies largely examined romantic relationships among adult samples or parent/child relationships among adolescent samples; one study examined peer relationships among adolescents. These studies are summarized in Table 3.

#### Daily

##### T1D.

Eight reports have examined daily links of relationship factors to glucose. Four of these were conducted among adults in romantic relationships and examined aspects of partner support provision. A small (*N* = 23) study examined both positive and negative aspects of support (Yorgason et al., 2023). Results showed that no support variable was linked to same-day glucose mean, but both patient and spouse reports of instrumental support predicted higher next-day glucose mean. For participants that wore a CGM (*N* = 14), links to TIR were also examined. These findings generally showed that emotional support was beneficial for TIR while controlling behavior, overprotective behavior, and instrumental support were detrimental. These results should be interpreted with caution given the small sample.

A second study examined diabetes-specific support/collaboration and illness shared appraisal (Berg et al., 2020). Results showed that PWDs’—but not partners’—daily perceptions of diabetes support/collaboration were associated with higher daily mean glucose. Illness shared appraisal (i.e., the extent to which each couple member viewed diabetes as shared) was not associated with glucose and did not interact with support/collaboration to predict glucose.

A similar study found that diabetes support from one’s partner was not associated with same-day glucose (Kent de Grey et al., 2022). However, partner diabetes support on one day predicted higher next-day glucose, but only among those in casual (versus committed) relationships. Thus, for this subgroup, diabetes support was detrimental for future glucose management.^3^ Another study from the same lab group combined partner and PWD reports of support to assess the implications of invisible support for glucose (Kent de Grey et al., 2021). Results showed invisible support from one’s partner was linked to lower same-day glucose mean, SD, and average daily risk ratio, but only for couples in which the partner reported high relationship satisfaction. Invisible support on one day also predicted lower mean glucose the next day, but again only when partners reported high relationship satisfaction.

The remaining four studies examined PWD/parent relationships. One study examined the effect of parental persuasive strategies around diabetes management on same-day and next-day glucose (Berg et al., 2013). Results showed no link of persuasive strategies and either mean glucose or glucose average daily risk range on the same day. However, mothers’, but not fathers’, use of persuasive strategies on one day predicted lower mean glucose the following day. Another study using the same sample examined parental conflict and found that conflict with both parents predicted lower mean glucose, greater risk for low glucose events, and lower risk for high glucose events on the same day (Campbell et al., 2023). Conflict did not predict next-day glucose. This study also assessed whether parental acceptance moderated the link of conflict to glucose; no evidence of moderation was found. Another study from the same lab examined adolescent diabetes disclosure to parents, as well as parents’ solicitation of diabetes information from their child (Berg et al., 2017). Findings showed that adolescent disclosure was unrelated to same-day glucose. Similarly, neither parents’ solicitation of diabetes information was linked to same-day glucose. A follow up study of the same sample one year later found that disclosure to fathers was associated with lower daily glucose, but only among PWD who still lived with their parents (Kelly et al., 2019). Disclosure to mothers, and solicitation of information by either parent, were unrelated to glucose.

##### T2D.

Only one study has examined daily links of social interactions to glucose among people with T2D. In this study, invisible social control was linked to improved same-day glucose mean, TIR and SD, but not glucose CV (Brownlee et al., 2024). These findings align with Kent de Grey et al., 2021, supporting the idea that partners’ supportive efforts may be most beneficial when provided invisibly.

##### Summary.

Together, this work suggests that support from romantic partners can both benefit and harm glucose at the daily level, that relationship-level factors play an important role in these processes, and that support that is provided invisibly may be most beneficial. Research on parent/adolescent interactions to glucose were similarly mixed, with greatest evidence for an effect of conflict on glucose. The wide range of independent variables examined in this literature—each of which likely impacts glucose in distinct ways—makes direct comparison of these findings challenging. These findings also highlight the complexity of glucose dynamics and the need to differentiate dangerous low glucose events from typically desirable reductions in mean glucose levels.

#### Within-day

##### T1D.

Three studies have examined within-day links of social interactions to glucose. The first investigated peer interactions among adolescents and found that neither interaction enjoyment nor upset since the prior prompt were linked to current glucose (Helgeson et al., 2009). Findings did not differ across males versus females. Another study from the same lab group, also among adolescents, found that experiencing a social interaction was associated with worse glucose SD, TIR, and TAR over the next two hours (Horner & Helgeson, 2024). Quality of the social interaction and interaction partner did not impact these findings. A final study examined whether socializing or caring for others^4^ prior to an EMA report were linked to current glucose readings; no links were found (Hernandez et al., 2023).

##### T2D.

Only one study has examined within-day links. This study found that partner involvement in one’s diabetes care predicted improved glucose outcomes over the next hour for five of the six glucose metrics examined (glucose mean, SD, and CV, TIR, and TAR; (Soriano et al., 2022). There was no link to TBR. Interestingly, the authors also examined the reverse temporal path, and found that only glucose mean and TBR predicted increased odds of partner involvement. Evidence was thus stronger in the direction of partner interactions predicting subsequent glucose outcomes.

##### Summary.

The within-person studies on social interactions provided conflicting evidence for links to glucose making it premature to draw conclusions. The differences may be explained by the different populations (T1D adolescents, T2D adults), or relationships (peers, partners) studied.

## Discussion

In the context of diabetes, one’s emotional experiences and social environment may have immediate implications for the state of one’s disease. Here, we presented a framework based on existing theory and research in medicine and social psychology that may explain these links. We then reviewed studies in this area that investigated how mood, stress, and social interactions within individuals impact short-term glucose outcomes over subsequent days or hours. While this literature is small and varied, several primary findings emerged across domains that have significant implications for future research in this area. Below, we consider our findings in the context of the proposed theoretical models.

There was somewhat consistent evidence that negative affect and stress are associated with negative within-person glucose outcomes, with most studies in these areas finding at least partial support. Similarly, negative social interactions (e.g., family conflict, controlling or overprotective behaviors) tended to be linked to poor glucose outcomes (Campbell et al., 2023; Yorgason et al., 2023) but not in the context of peer relationships (Helgeson et al., 2009). These findings are consistent with the model presented in Fig. 1, although the mechanisms underlying these links were rarely investigated.

Considering the effects of positive experiences on glucose, the findings were more mixed. For positive affect, about half of the studies found at least partial support for a link to positive outcomes. These inconsistent results may reflect our hypothesis that the effect of positive affect depends on stress. That is, positive affect will do little to impact the physiological pathway if the physiological stress response is not already activated. Similarly, positive affect may be most beneficial for health behaviors as a buffer against stressful experiences, allowing one to reappraise available resources or increase problem-solving and coping strategies. While some research has investigated this hypothesis at the between-person level (Horner et al., 2023), this has not been examined in intensive longitudinal contexts.

Social interactions that would generally be considered positive, like social support, also showed contradictory findings in relation to glucose. These interactions were linked to both negative (Berg et al., 2020b; Kent de Grey et al., 2022; Yorgason et al., 2023) and positive (Kent de Grey et al., 2021; Yorgason et al., 2023) glucose outcomes. Similarly, social interactions that were not clearly positive or negative (e.g., parents’ persuasive strategies, partner involvement, disclosure/solicitation of diabetes information) showed mixed results (Berg et al., 2013, 2017; Soriano et al., 2022). These contradictory findings may point to the hypothesized role of self-determination needs as presented in Fig. 2. There is a great deal of research on how well-intended support can be perceived as intrusive or controlling or can otherwise go awry, and diabetes is no exception to this phenomenon (Lewis & Rook, 1999; Van Vleet & Helgeson, 2020). Self-determination theory offers a framework to understand the contexts in which social interactions may be perceived as beneficial versus detrimental. That is, social interactions that support these needs should improve outcomes, while social interactions that undermine these needs should harm outcomes. Future work should investigate these moderated pathways.

A notable limitation to the extant literature is that the mechanisms underlying the links of psychosocial processes to glucose, depicted in Figs. 1 and 2, are rarely assessed. The one study in the present review that conducted within-person mediation did so by examining a construct relevant to the behavioral pathway of our proposed models. The study examined diabetes task competence, a combination of perceived self-efficacy related to diabetes and the execution of disease management behaviors. Results showed that daily diabetes task competence fully mediated the link between both negative and positive affect and same-day glucose (Fortenberry et al., 2009). This study provides support for disease management behaviors as a mechanism linking psychosocial factors to physiological disease markers. However, the cross-sectional nature of this finding precludes interpretation of the temporal ordering of these links, and the collapse of two constructs (self-efficacy and behaviors) into one muddies the interpretation of these processes. Additionally, this study assessed daily links and thus did not clarify the timescale through which such processes may play out within a single day. While the directional link between related psychosocial variables (i.e., mood and self-efficacy in the present study) may be a valuable line of future research, first identifying the mechanisms that link psychosocial factors, broadly speaking, directly to physiological changes may better facilitate applications of this work.

A number of studies have independently investigated links of psychosocial factors to self-care behaviors or self-care to glucose, thus providing indirect support for this mechanistic link. For example, in addition to assessing links of psychosocial factors to glucose, studies in the present review found disclosure to mothers (but not fathers) predicted better same-day adherence among adolescents with T1D (Berg et al., 2017), and diabetes stressors were inversely related to same-day self-care (Berg et al., 2020a, b; Kelly et al., 2020; Kent de Grey et al., 2022). Studies outside of the present review confirm the link of social or psychological experiences to self-care (Fortenberry et al., 2012; Gonzalez et al., 2007; Schmitt et al., 2021; Wiebe et al., 2016) although it should be noted that positive events and moods have been linked to both better and worse self-care behaviors (Mulvaney et al., 2019). From the other direction, it is well established that self-care behaviors are central to the regulation of glucose. One study in the present review found that self-care activities predicted lower glucose values (including *increased* risk for hypoglycemia) across the subsequent 10 h, controlling for negative and positive affect (Wagner et al., 2017). Other research using EMA or daily diary techniques have confirmed the link of self-care behaviors to improved glucose outcomes at the within-person level (Tracy et al., 2019, 2020).

It should be noted that in measuring diabetes stressors, several studies included items that inherently capture self-care behaviors (e.g., “forget or skip a blood glucose test”; Berg et al., 2020a; Kelly et al., 2020; Kent de Grey et al., 2022). While maintenance of a complex regimen is one of the key disease stressors experienced by PWD (Fisher et al., 2015), conflating stress with self-care failures is problematic in the context of the proposed theoretical models because it muddies the distinction between predictor and hypothesized mechanism. Future evaluations of these mechanisms should take care to differentiate health behaviors from the subjective experience of stress.

It is important to underscore that the total number of studies in each area is quite small, and there is considerable heterogeneity across study foci and methodologies. For example, studies differed in terms of theoretical orientation, independent variable conceptualization, glucose outcome metric, population (e.g., age, disease type), and analysis plan. This was especially true of the social interaction literature, where relationship type and predictor variables varied widely. It is therefore challenging to make broad conclusions that apply across this developing literature. There is also considerable imbalance in the reviewed studies. For example, only five studies identified by this review were conducted among people with T2D—none of which investigated stress. Thus, the conclusions identified here should be interpreted with caution, subject to future replications, and examined across contexts and disease types.

### Current limitations and guidelines for future research

While the present review identified a budding literature on the psychosocial predictors of short-term glucose, several key limitations emerged. Some of these limitations simply reflect the early stage of this literature. Having few total studies as well as great heterogeneity (e.g., different disease types, sample ages, construct of interest) means that studies cannot easily be compared to each other. Thus, there is a need for overall greater work in this area. However, other limitations emerged that deserve more careful attention in the future. Below, we summarize these limitations as well as our recommendations for addressing each. Our aim is to support methodologically robust future research to progress our understanding of the real-time implications of psychosocial factors for glucose.

#### 1. Design studies to explicitly test and compare the directionality of the hypothesized links.

The majority of studies on the links of mood and stress to glucose examined contemporaneous rather than lagged links and thus do not speak to the direction of these relations. It is certainly the case that glucose excursions can impact psychosocial experiences rather than vice versa. Indeed, several studies reviewed here approached this question from this direction (e.g., Henriksen et al., 2021; Polonsky & Fortmann, 2020; Williams et al., 2002) but conducted contemporaneous analyses. Future studies must move beyond contemporaneous or cross-sectional analyses to probe the direction of these relations, ideally by testing cross-lagged links in the same models. (See Soriano et al. (2022) for a thoughtful example.) Doing so will allow for stronger specification of causal models and clearer interpretation of extant findings. It is noteworthy that the social interaction literature frequently tested lagged links and typically found greater evidence for lagged versus concurrent relations.

#### 2. Design studies to explicitly test the timescale and duration of the hypothesized links.

A second issue related to timing is the overemphasis on daily compared to within-day links; this is especially true in the stress and social interaction literatures. Only nine studies in the present review investigated how psychosocial factors impact glycemia within the course of a single day, most of which assessed contemporaneous links. Considering the intersection of points (1) and (2), care must be taken in considering the time scale of lags. Daily lags may not be of sufficient temporal resolution to tease apart the mechanisms in question. For example, the physiological stress response onsets within about 15 min, and disruptions to self-care (e.g., forgetting to eat) would impact glucose over a period of hours, not days. Future work should focus on mapping these links at a finer temporal resolution.

As noted, lags were more frequently assessed in the social interaction literature and there was often evidence for next-day effects of social interactions on glucose. While lack of within-day lagged research is still a limitation of this literature, these day-level lagged findings suggest that it may take time to absorb or internalize a social interaction and that the impact on one’s diabetes is thus delayed.

#### 3. Evaluate hypothesized mechanisms underlying the hypothesized links.

The proposed psychological, physiological, and behavioral pathways should be explicitly evaluated. For example, ambulatory heart rate monitors, bolus information from insulin pumps, or self-reported disease management behaviors could be paired with psychosocial EMA and CGM data to evaluate mechanistic models. Importantly, autonomy, competence, and relatedness could be measured alongside stress, mood, and social interactions to better understand how these experiences influence glucose. Related to the previous guideline, it is likely that the proposed physiological pathway operates on a shorter timescale compared to the behavioral pathway; these mechanistic differences must be considered in designing studies to evaluate these paths.

#### 4. Appropriately account for other factors that influence glucose levels.

Food intake, activity levels, and medication adherence/insulin administration bear great influence on one’s glucose levels. In identifying the unique effect of psychosocial factors on glucose, these additional variables must be taken into consideration. However, doing so may complicate interpretation to the extent that these factors overlap with the hypothesized behavioral pathway.

#### 5. Consider more carefully the glucose outcomes of interest.

In the present review, many studies (particularly those that used CGMs) tested a large number of glucose outcomes, with no theoretical predictions as to which ones would be most impacted by a given psychosocial predictor or mechanistic pathway. Given the early stage of this literature, this exploratory approach is understandable. However, moving forward the literature would benefit from more careful consideration of outcome measures so that statistical errors associated with multiple testing can be avoided. For example, there was some evidence in the present review that mood tended to be unrelated to glucose variability, a finding shared by a prior review that included between-person research (Muijs et al., 2021). Such findings should be interrogated with both theory and replication. This issue is compounded by the fact that CGM metrics are typically validated at the daily, not within-day level; their clinical meaning over these short periods is not clear. For example, while international guidelines recommend spending at least 70% of one’s day in range (Danne et al., 2017), it is not clear that the same 70% target should be applied to each individual hour throughout the day. Validation of these outcomes combined with stronger theory will produce more robust hypotheses and reduce statistical error associated with multiple testing.

Finally, many studies in this review used blinded CGM devices in which participants are not able to see their glucose readings. Blinding is useful when one wishes to control for participants’ reactions to glucose numbers. However, given the substantial increases in CGM use, (Karakus et al., 2023), use of blinded sensors may no longer be ecologically valid. To fully understand how glucose interacts with psychosocial experiences, research needs to account for the fact that many PWD are constantly checking—and reacting to—their glucose numbers. On the one hand, the burden associated with such continual feedback may negatively impact PWD. On the other hand, receiving such detailed and timely information may allow PWD to successfully respond to glucose excursions quickly. Thus, the experiences of CGM use—and diabetes technology more broadly—should be included in both theory and methodology aimed at understanding the daily experiences of PWD.

#### 6. Conduct more research among people with T2D.

The majority of research identified in the present review was conducted among people with T1D. Given that T1D and T2D involve different pathophysiologies and behavioral management regimens, it is likely that they also differ in terms of the effects of psychosocial factors on glucose. Greater investigation among those with T2D, or inclusion of disease type as a moderator of the hypothesized links, will help clarify these differences and may point to distinct mechanistic pathways or timescales for each disease.

#### 7. Further investigate potential moderators of the psychosocial factor-glucose link.

Situational or person-level factors may impact the way or extent to which psychosocial factors operate on glucose. Indeed, many findings in the social interaction literature depended on person- or relationship-level moderators like gender, relationship satisfaction, or type of relationship. Of these, future research should pay particular attention to gender. Prior work at the between-person level has found gender to impact the extent to which psychosocial factors impact disease and well-being outcomes among PWD, but the direction varies across studies (Helgeson et al., 2023; Van Vleet & Helgeson, 2020). If gender is a significant moderator, future work should investigate whether findings are the result of biological differences in physiological responses to emotions or stress (Goel et al., 2021) or stem from differences in the socialization of men and women.

## Supplementary Material

Supplement

## Figures and Tables

**Table 1:** Studies assessing within-person associations of mood and affect to short-term glucose

1st Author, Year	Sample	Study length	Included lagged	Psychosocial Variable(s)	Glucose Device: Metric(s)	Covariates	Relevant findings

***Daily Links***							
*Type 1 diabetes*							
de Wit, 2023	18 adults 56% female 100% White Country: Netherlands	14 days	No	Anxiety, depression, anger, fatigue, vigor measured six times per day. Mean and CV of each mood calculated across the day	CGM (blinded): Daily (8 AM-6 PM) CV	Day of week, Sleep duration, Sleep quality, Nocturnal hypoglycemia	Overall, daily glucose CV not associated with mean or CV of mood ratings. However, on days following a nocturnal hypoglycemia event, glucose CV associated with mean mood (more anxiety, depressed mood, and fatigue; less vigor) and higher mood CV (anger, vigor). On days with glucose CV > 36%, participants had greater fatigue CV and vigor CV. Sleep quality or duration did not moderate findings
Fortenberry, 2009	62 adolescents 53% male 92% Caucasian Country: United States	14 days	Yes	Daily negative and positive affect	SMBG: Daily mean	Prior timepoint dependent variable (lagged models only), day of study	Negative affect associated with higher same-day mean glucose. Positive affect associated with lower same-day mean glucose. Neither negative nor positive affect predicted next-day mean glucose
Lansing, 2017*	180 adolescents 46% male 95% Caucasian Country: United States	14 days	No	Daily negative affect about diabetes	SMBG: Daily mean and SD	Age, time since diagnosis	Negative affect associated with same-day mean glucose and glucose SD
Polonsky, 2020	219 adults with self-reported high glucose variability 31.5% male 89% non-Hispanic white Country: Not reported (online recruitment)	14 days	No	Daily ratings of individual mood items Positive: cheerful, full of energy, “calm and relaxed”, overall mood Negative: frustrated, irritable, anxious, sad, exhausted, difficulty concentrating	CGM (not blinded): Daily TIR, TAR (>300 mg/dL), TBR, and CV	Well-being, Perceived Stress, Diabetes Distress	All negative items individually associated with lower TIR except “sad”, all negative items individually associated with greater TAR except “exhausted” and “irritable”. Only “exhausted” associated with CV. Negative affect not associated with TBR. All positive items individually associated with greater TIR; all positive items except “calm and relaxed” associated with TAR. Positive affect not associated with CV or TBR
Shapira, 2021	32 teens 56% female Race/ethnicity not reported Country: United States	14 days	No	Daily negative and positive affect	SMBG: Daily CV	Age, sex, diabetes duration, pump use	Negative and positive affect not associated with daily CV
*Type 2 diabetes*							
Skaff, 2009	206 adults 41.3% male 37.9% European-American, 22.8% African-American, 20.4% Asian, 10.2% Latino, 8.7% other Country: United States	21 days	Yes	Daily negative and positive affect	SMBG: Fasting morning glucose	Gender, age, education, ethnicity, BMI, time with diabetes, insulin use, MDD, depressed mood, HbA1c, HbA1c^2^ Positive and negative affect included as simultaneous predictors	Negative affect on one day predicted next-morning fasting glucose. Exploratory analyses showed this effect was only significant among men. Positive affect did not predict next-morning fasting glucose
**Within-Day Links**							
*Type 1 Diabetes*							
de Wit, 2023	18 adults 56% female 100% White Country: Netherlands	14 days, 6 EMAs per day	No	Momentary anxiety, depressive mood, anger, fatigue, vigor. Six measures per day	CGM (blinded): Momentary glucose levels categorized as below range (< 4 mmol/l), in-range (4– 10 mmol/l), above range (>10 mmol/l)	Day of week	Above-range momentary glucose values associated with higher anger and lower vigor
Hermanns, 2007	36 adults 77.8% male Race/ethnicity not reported Country: Germany	Mean = 48.8 h, Mean = 15.7 EMAs total	No	Momentary mood: Hedonic tone, energetic arousal, tension, anger	CGM (blinded): Momentary glucose value,	Number of mood ratings, insulin use, time of day	Tension associated with greater momentary glucose. Hedonic tone and energetic arousal associated with lower momentary glucose. No associations with anger
Horner, 2024	88 adolescents 53.4% male 94.3% non-Hispanic White, 4.5% Black, 1.1% Asian Country: United States	8 days, 8 EMAs per day	Yes	Positive and negative affect since prior EMA survey	CGM (not blinded): mean, TIR, TBR, SD for the two hours following each EMA survey	Prior timepoint dependent variable, exercise, eating, weekday vs. weekend, fall vs. spring	No associations between negative or positive affect and glucose
Shapira, 2021	32 teens 56% female Race/ethnicity not reported Country: United States	14 days, 4 EMAs per day	No	Momentary negative and positive affect	SMBG: Momentary glucose classified as: In-range, high (> 180 mg/ dL), very high (>250 mg/dL), low (<70 mg/ dL), very low (<54 mg/dL)	Age, sex, diabetes duration, pump use	Positive affect associated with greater odds of in-range glucose and reduced odds of very low glucose, only among those with HbA1c ≤ 8% Negative affect associated with greater odds of very high (>250 mg/dL) glucose, especially for those with HbA1c ≤ 8%
*Type 2 Diabetes*							
Wagner, 2017	50 adults 26% male 100% Latino Country: United States	7 days, 2 measures per day	Yes	Negative and positive affect measured morning and night	CGM (blinded): Mean, SD, %TBR, %TAR, %TOR across 2- and 10-hour bins	Prior timepoint dependent variable, time of assessment (AM/PM), medication adherence Positive and negative affect, self-care behaviors included as simultaneous predictors	No links of negative or positive affect to subsequent glucose outcomes
Williams, 2002	94 adults 56% male 87% Caucasian, 12% African American, 1% Asian Country: United States	7 days, 4 EMAs per day	No	Momentary negative and positive affect	SMBG: Momentary value	Time of assessment, neuroticism	No link between glucose and negative affect; neuroticism did not moderate link of glucose to negative affect. A quadratic glucose term showed that high and low glucose were associated with greater negative affect. No link between glucose, quadratic glucose term, and positive affect. Neuroticism did not moderate link of glucose to positive affect

*Note:* Studies that examined both daily and within-day links are presented twice for clarity. BMI = body mass index, CV = coefficient of variation, MDD = major depressive disorder, SD = standard deviation, SMBG = self-monitoring of blood glucose, TAR = time above range, TBR = time below range, TIR = time in range, TOR = time out of range

* Berg et al., 2013, Lansing et al., 2017, and Campbell et al., 2023 used data from the same sample

**Table 2:** Studies assessing within-person associations of stress and short-term glucose

1st author, year	Sample	Study length	Included lagged	Psycho-social variable(s)	Glucose Device: Metric(s)	Covariates	Relevant findings

***Daily Links***							
*Type 1 Diabetes*							
Benjamin, 2021	44 adolescents 68.2% female 68.6% White; 13.7% Hispanic, 9.8% Asian; 3.9% Black, 11.8% Other Country: United States	7 days	No	Daily general stress and diabetes stress	SMBG/CGM (not blinded): Daily mean and SD	Gender, age at diagnosis, trait anxiety	Daily diabetes stress associated with higher same-day mean glucose; this link was not moderated by trait anxiety. No association between daily diabetes stress and same-day glucose SD. No association between daily general stress and same-day glucose mean or SD
Berg, 2020a†	199 adults 52.3% female 92.5% White 6% Hispanic Country: United States	14 days	No	Daily general stressors and diabetes stressors	SMBG: Daily mean	Day, age, number of comorbidities	Daily diabetes stressors associated with higher same-day glucose mean. This link was not moderated by comorbidities. Daily general stressors were not linked to same-day glucose mean. However, there was an interaction with comorbidities such that the link of general stressors to glucose mean was positive for those with more comorbidities and negative for those with fewer comorbidities Additional interactions were all n.s. (age, age*comorbidities, age*comorbidities*stress, and general*diabetes stress)
Berg, 2023††	207 emerging adults 65% female 75.2% non-Hispanic White 14.2% Hispanic 4.8% African American Country: United States	14 days	No	Daily general stressors and diabetes stressors	SMBG: Daily mean	Day, perceived stress, pump status	Daily diabetes stressors predicted higher same-day mean glucose. Daily general stressors did not predict same-day mean glucose. General stress did not interact with diabetes-specific stress to predict glucose. Perceived stress did not interact with general or diabetes-specific stress to predict glucose
Ehrmann, 2022	178 adults recruited from inpatient setting 58% female Race not reported Country: Germany	17 days	No	Daily diabetes distress, Hypoglycemia distress, Hyper-glycemia distress, Glucose variability distress	CGM (not blinded): Daily TBR, TAR, CV	Gender, diabetes distress, depressive symptoms, number of CGM scans, study day	Daily TBR associated with greater same-day hypoglycemia distress and less same-day hyperglycemia distress. No link to overall diabetes distress or variability distress. Daily TAR associated with greater diabetes distress, hyperglycemia distress, and variability distress, and less hypoglycemia distress. Daily glucose CV associated with greater diabetes distress, hypoglycemia distress, hyperglycemia distress, and variability distress
Gonder-Frederick, 2016‡	33 adults 51.5% female All White non-Hispanic Country: United States	7-15 Days (M = 8.9, SD = 1.8)	No	Single item of general daily stress	CGM (partial blinded), daily summaries: Mean, TBR, TAR, AUC, low and high glucose risk indices, SD, glucose risk index, risk range, glucose rate of change SD	Participant, carbohydrate intake, daily insulin	Daily stress associated with greater same-day TBR, SD, daily risk index, daily risk range, and glucose rate of change. No association with glucose mean, TAR, AUC, low and high-risk indices, or daily risk index
Ozaslan, 2018‡	37 adults Gender not reported Race/ethnicity not reported Country: United States	7 days	No	Single item of general daily stress	CGM (blinded): Daily mean, effectiveness index	BMI	Stress not associated with glucose mean. Stress associated with glucose effectiveness index, but only for those with high BMI

*Note*: No studies assessed within-person links of stress to glucose; no studies assess links of stress to glucose among people with T2D. AUC = area under the curve, BMI = body mass index, CV = coefficient of variation, SD = standard deviation, SMBG = self-monitoring of blood glucose, TAR = time above range, TBR = time below range

† Berg et al., 2020a, 2020b, and Kent de Grey et al., 2021 used data from the same sample

†† Berg et al., 2017, Kelly et al., 2019, Kent de Grey at al., 2022, and Berg et al., 2023 used data from the same sample

‡ Gonder-Frederick, 2016 and Ozaslan, 2018 used data from the same study

**Table 3:** Studies assessing within-person associations of social interactions and short-term glucose

1st author, year	Sample^+^	Study length	Included lagged analysis	Psychosocial variable(s)	Glucose Device: Metric(s)	Covariates	Relevant findings

***Daily Links***							
*Type 1 diabetes*							
Berg, 2013*	180 adolescents and their parents 54.4% female 94.9% Caucasian Country: United States	14 days	Yes	Parental persuasive strategies	SMBG: Daily mean, average daily risk range	Prior timepoint dependent variable (lagged models only), day, number of diabetes problems	Daily glucose mean and risk range not associated with same-day parental persuasive strategies. Mothers’, but not fathers’ use of persuasive strategies predicted lower next-day mean glucose
Berg, 2017††	236 adolescents 62% female 75.2% White, 14.2% Hispanic, 4.8% African American Country: United States	14 days	No	Diabetes disclosure to mother/father, Mother/father diabetes solicitation	SMBG: Mean	Day, pump status, years since diagnosis Mother and father disclosure and solicitation included in same model	Daily solicitation and disclosure to mothers and fathers was not associated with daily mean glucose
Berg, 2020b†	199 adults and their romantic partners 52.3% female 89.9% non-Hispanic While Country: United States	14 days	No	Perceptions of partner’s collaboration/support, illness appraisal (shared vs. not shared)	SMBG: Mean	Study site, day, relationship quality, gender, length of diagnosis, pump status	PWDs’ daily perceptions of partners’ collaboration/support associated with higher daily mean glucose. Partners’ perceptions of own collaboration/ support not associated with PWDs’ daily mean glucose. Neither partner nor PWD illness appraisal associated with glucose. Collaboration/support did not interact with illness appraisal to predict glucose
Campbell, 2023*	180 adolescents 54% female 95% White Country: United States	14 days	Yes	Family conflict	SMBG: Mean, high and low risk indices (exploratory)	Prior timepoint dependent variable (lagged models only), day, pump status, sex, age, parental acceptance	Daily conflict with both mothers and fathers associated with lower same-day mean glucose. Exploratory analyses showed conflict was associated with greater risk for low glucose episodes, and lower risk for high glucose episodes. Parental conflict on one day did not predict next-day glucose. Daily perceptions of parental acceptance did not moderate link of conflict to glucose
Kelly, 2019††	212 emerging adults 66.4% female 76.26% White, 5.05% Black, 2.53% Other race, 2.53% multiple races; 13.36% Hispanic Country: United states	14 days	No	Diabetes disclosure to mother/father, mother/father diabetes solicitation	SMBG: Mean	Living with/without parents, insulin pump, gender, day	Daily disclosure to mothers was unrelated to blood glucose regardless of whether the PWD lived with parents or not. Daily disclosure to fathers was linked to lower same-day glucose, only among PWD who lived with parents Daily diabetes solicitation by mothers or fathers was unrelated to glucose, regardless of living situation
Kent de Grey, 2021†	199 adults and their romantic partners 52.3% female 92.5% White; 6% Hispanic Country: United States	14 days	Yes	Support visibility	SMBG: Mean, SD, average daily risk range (ADRR)	Prior timepoint dependent variable (lagged models only), gender, age, relationship satisfaction	Invisible support associated with lower same-day and next-day mean glucose, only for couples with high partner relationship satisfaction. Invisible support linked to lower same-day glucose SD and ADRR, only for couples with high partner relationship satisfaction. No links of invisible support to next-day glucose SD or ADRR
Kent de Grey, 2022††	101 emerging adults in a romantic relationship 28.7% male Race/ethnicity not reported Country: United States	14 days	Yes	Diabetes support from romantic partner	SMBG: Mean	Prior timepoint dependent variable (lagged models only), day, pump status, years since diagnosis, diabetes stressors, relationship type (casual vs. committed)	Partner diabetes support on one day not associated with glucose mean on that day. Partner diabetes support on one day predicted higher next-day mean glucose, only among those in casual (versus committed) relationships
Yorgason, 2023	23 heterosexual married couples 52.2% female 77.8% White, 22.2% Hispanic Country: United States	9-10 days	Yes	Patient and spouse reports of emotional support, instrumental support, overprotective behavior, illness avoidance, and controlling behavior	SMBG / CGM (not blinded): Mean, TIR only for CGM participants	Gender, number of children, education, income, time since diagnosis, pump status, day	In fully adjusted models, no same-day links of any support variable to daily glucose mean Spouse daily controlling, overprotective, and instrumental support linked to lower same-day TIR; spouse within-person emotional support linked to higher same-day TIR. Both patient and spouse instrumental support on one day predicted higher next-day glucose mean. Spouse daily controlling behavior predicted higher next-day TIR
*Type 2 diabetes*							
Brownlee, 2024§	63 adult PWD and their romantic partners 64% male 70% White, 24%, Black, 5% multiracial/other, 2% Asian; 97% non-Hispanic Country: United States	8 days	No	Invisible social control	CGM (blind): mean, TIR, SD, CV	PWD and partner reports of diabetes social control	Invisible social control associated with improved same-day glucose mean, TIR, and SD. There was no link of social control to glucose CV
***Within-Day Links***							
*Type 1 diabetes*							
Helgeson, 2009	76 adolescents 50% female 89.5% Caucasian; 9.2% African American, 1.3% Hispanic Country: United States	4 days, 6-8 EMAs per day	Yes	Peer interaction enjoyment, Peer interaction upset	SMBG: Momentary readings	Gender	Interaction enjoyment or upset (reported since prior EMA signal) was not linked to glucose measured at current EMA signal. Gender did not moderate these links
Hernandez, 2023	92 adults 53% female 34% White, 35% Latino, 18% African American, 4% multiethnic, 3% Asian, 2% other, 3% not reported Country: United States	14 days, up to 6 EMAs per day	No	Socializing, caring for others	CGM (not blinded): momentary reading within 15 min of EMA	Dummy variables indicating set of daily activities (e.g., working, traveling); time of day, day of week, age, gender, ethnicity, income	“Socializing” was not associated with momentary glucose. “Caring for others” was not associated with momentary glucose
Horner, 2024	88 adolescents 53.4% male 94.3% non-Hispanic White, 4.5% Black, 1.1% Asian Country: United States	8 days, 8 EMAs per day	Yes	Social interaction, emotional support, conflict, communality, dominance, unmitigated communality	CGM (not blinded): mean, TIR, TAR, SD for the two hours following each EMA	Prior timepoint dependent variable, exercise, eating, weekday vs. weekend, fall vs. spring	Experiencing a social interaction at one EMA predicted higher glucose SD and lower odds of achieving TIR and TAR goals over the next two hours. Emotional support, conflict, communality, dominance, or unmitigated communality during social interaction did not further predict glucose outcomes
*Type 2 Diabetes*							
Soriano, 2022§	63 adult PWD and their romantic partners 63.5% male 95.2% non-Hispanic; 69.8% White; 23.8% Black, 1.6% Asian, 4.8% Other Country: United States	7 days, 5 EMAs per day	Yes	Partner involvement in self-care	CGM (blinded): Mean, SD, CV, TIR, TAR, TBR	Prior timepoint independent variable, hours since study start, time of day	Partner involvement predicted glucose mean, SD, CV, TIR, and TAR over the next hour. Partner involvement did not predict next-hour TBR

*Note*: ADRR = average daily risk range, CV = coefficient of variation, SD = standard deviation, SMBG = self-monitoring of blood glucose, TAR = time above range, TBR = time below range, TIR = time in range

+ Demographics refer to PWD

* Berg et al., 2013, Lansing et al., 2017, and Campbell et al., 2023 used data from the same sample

† Berg et al., 2020a, Berg et al., 2020b, and Kent de Grey et al., 2021 used data from the same sample

†† Berg et al., 2017, Kelly et al., 2019, Kent de Grey at al., 2022, and Berg et al., 2023 used data from the same sample

§ Brownlee et al., 2024 and Soriano et al., 2022 used data from the same sample

## Footnotes

1 Another study used these same data, including the same measures of diabetes stressors and glucose, to understand the role of attachment and support in diabetes-related stress (Kelly et al., 2020). This study is not included in this review because investigations of stress and support in this sample are more directly evaluated in, Berg et al., 2020a and 2020b, respectively

2 Another study used these same data, including the same measures of diabetes stressors and glucose, to understand the impact of executive functioning on diabetes management (Wiebe et al., 2023). This study is not included in this review because the link of stress and glucose in this sample is more directly evaluated in Berg et al., 2023.

3 Number and severity of diabetes stressors were included in these models as controls. These findings are not discussed because the links of stress to glucose in this sample were directly investigated in Berg et al., 2023.

4 “Caring for others” included some non-social activities, like caring for pets.
