# Supplementary material for: Psychosocial predictors of short-term glucose among people with diabetes: A narrative review
Source: J Behav Med. Author manuscript; Available in PMC 2025 Apr 1. (PMC11929727; doi:10.1007/s10865-024-00536-9)

Supplement 1: Literature Search Terms

PsychInfo:

AB ("Blood glucose" OR CGM OR continuous glucose monitor* OR "finger prick" OR glucometer OR "blood glucose self monitoring" OR Dexcom OR "FreeStyle Libre" OR Medtronic ) AND AB ( mood OR emotion* OR stress OR distress OR psychosocial OR "social relationships" OR "romantic relationships" OR "close relationships" OR "social interactions" OR "relationship quality" OR depress* OR anxiety OR anxious OR anger OR angry OR happy OR "well being" OR "wellbeing" OR "well-being" OR "positive affect" OR "negative affect" OR "social support" ) AND AB ( daily OR diary OR day OR days OR "24 h" OR "ecological momentary assessment" OR "experience sampling" OR "ambulatory assessment" ) AND AB diabetes NOT ( mice OR rats OR rodents OR animals OR bovine)

PubMed:

(((("blood glucose"[Title/Abstract] OR CGM[Title/Abstract] OR continuous glucose monitor*[Title/Abstract] OR "finger prick"[Title/Abstract] OR glucometer[Title/Abstract] OR "blood glucose self monitoring"[Title/Abstract] OR Dexcom[Title/Abstract] OR "FreeStyle Libre"[Title/Abstract] OR Medtronic[Title/Abstract]) AND (mood[Title/Abstract] OR emotion*[Title/Abstract] OR stress[Title/Abstract] OR distress[Title/Abstract] OR psychosocial[Title/Abstract] OR "social relationships"[Title/Abstract] OR "romantic relationships"[Title/Abstract] OR "close relationships"[Title/Abstract] OR "social interactions"[Title/Abstract] OR "relationship quality"[Title/Abstract] OR depress*[Title/Abstract] OR anxiety[Title/Abstract] OR anxious[Title/Abstract] OR anger[Title/Abstract] OR angry[Title/Abstract] OR happy[Title/Abstract] OR "well being"[Title/Abstract] OR "wellbeing"[Title/Abstract] OR "well-being"[Title/Abstract] OR "positive affect"[Title/Abstract] OR "negative affect"[Title/Abstract] OR "social support"[Title/Abstract])) AND (daily[Title/Abstract] OR diary[Title/Abstract] OR day[Title/Abstract] OR days[Title/Abstract] OR "24 h"[Title/Abstract] OR "ecological momentary assessment"[Title/Abstract] OR "experience sampling"[Title/Abstract] OR "ambulatory assessment"[Title/Abstract])) AND (diabetes[Title/Abstract])) NOT (mice[Title/Abstract] OR rats[Title/Abstract] OR rodents[Title/Abstract] OR animal[Title/Abstract] OR bovine[Title/Abstract])

Web of Science:

((((AB=("blood glucose" OR CGM OR continuous glucose monitor* OR "finger prick" OR glucometer OR "blood glucose self monitoring" OR Dexcom OR "FreeStyle Libre" OR Medtronic)) AND AB=(mood OR emotion* OR stress OR distress OR psychosocial OR "social relationships" OR "romantic relationships" OR "close relationships" OR "social interactions" OR "relationship quality" OR depress* OR anxiety OR anxious OR anger OR angry OR happy OR "well being" OR "wellbeing" OR "well-being" OR "positive affect" OR "negative affect" OR "social support")) AND AB=(daily OR diary OR day OR days OR "24 h" OR "ecological momentary assessment" OR "experience sampling" OR "ambulatory assessment")) AND AB=(diabetes)) NOT AB=(mice OR rats OR rodents OR animal OR bovine)

Supplementary Figure 1: Flowchart of article selection process.


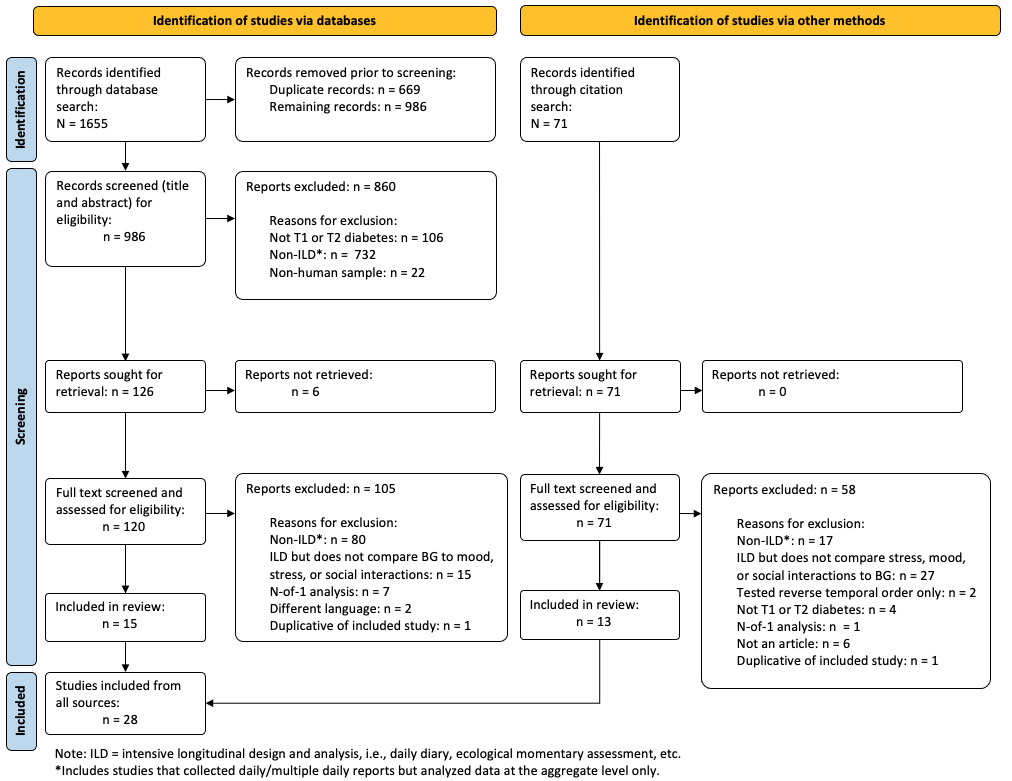

Supplement: Supplement [file NIHMS2047074-supplement-Supplement.docx]
